# Piezo2 in Mechanosensory Biology: From Physiological Homeostasis to Disease‐Promoting Mechanisms

**DOI:** 10.1111/cpr.70112

**Published:** 2025-08-09

**Authors:** Zhebin Cheng, Zuping Wu, Mengjie Wu, Liang Xie, Qianming Chen

**Affiliations:** ^1^ Stomatology Hospital, School of Stomatology, Zhejiang University School of Medicine, Zhejiang Provincial Clinical Research Center for Oral Diseases, Key Laboratory of Oral Biomedical Research of Zhejiang Province Engineering Research Center of Oral Biomaterials and Devices of Zhejiang Province Hangzhou China; ^2^ The Central Laboratory Peking University School and Hospital of Stomatology Beijing China

**Keywords:** mechanosensation, mechanosensory modality, organ system, pathogenesis, Piezo2

## Abstract

Piezo2, a mechanically activated ion channel, serves as the key molecular transducer for touch, proprioception and visceral sensation. These mechanosensation processes, where mechanical forces are converted into electrochemical signals, are essential for sensory perception, interoception and systemic homeostasis. Critically, Piezo2 channels are fundamental to diverse physiological functions, such as skeletal growth, respiratory development and inter‐organ homeostasis. Despite its established role in sensory neurons and specialised mechanotransducers, the molecular intricacy of Piezo2‐mediated signalling and its pathophysiological relevance remain incompletely understood. This review highlights key evidence from recent studies employing advanced technologies supporting the potential of Piezo2 channels as vital mechanosensor that regulate mechanotransduction cascades in physiological systems, demonstrating their potential as drug targets for the development of therapeutic agents.

## Graphical abstract

Piezo2 channels are essential mechanotransducers regulating touch, proprioception and visceral mechanosensation across physiological systems, emerging as therapeutic targets for pathological mechanical hypersensitivity and neurogenic disorders.

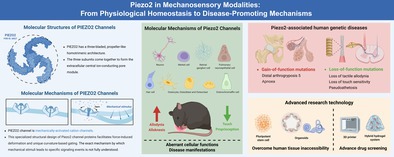

## 1 Introduction

Mechanosensation, the process by which cells detect and transduce dynamic mechanical perturbations into electrical and biochemical signals [1, 2], is crucial for humans and other animals to sense and respond to external and internal stimuli [3, 4, 5]. This process, spanning from cells to tissues, includes conscious touch perception (somatosensation), proprioception and postural control and autonomic regulation of vital physiological functions, such as hearing and blood pressure regulation (interoception) [2, 6, 7]. Mechanically activated (MA) ion channels represent a specialised class of mechanotransducers that mediate rapid cellular responses to mechanical stimuli through membrane depolarisation and/or Ca^2+^ signalling [8, 9]. However, their molecular identities have remained elusive for decades, impeding our comprehension of their physiological significance in vivo and the molecular mechanisms underlying the conversion of force sensing to selective cation permeation.

**FIGURE 1:**
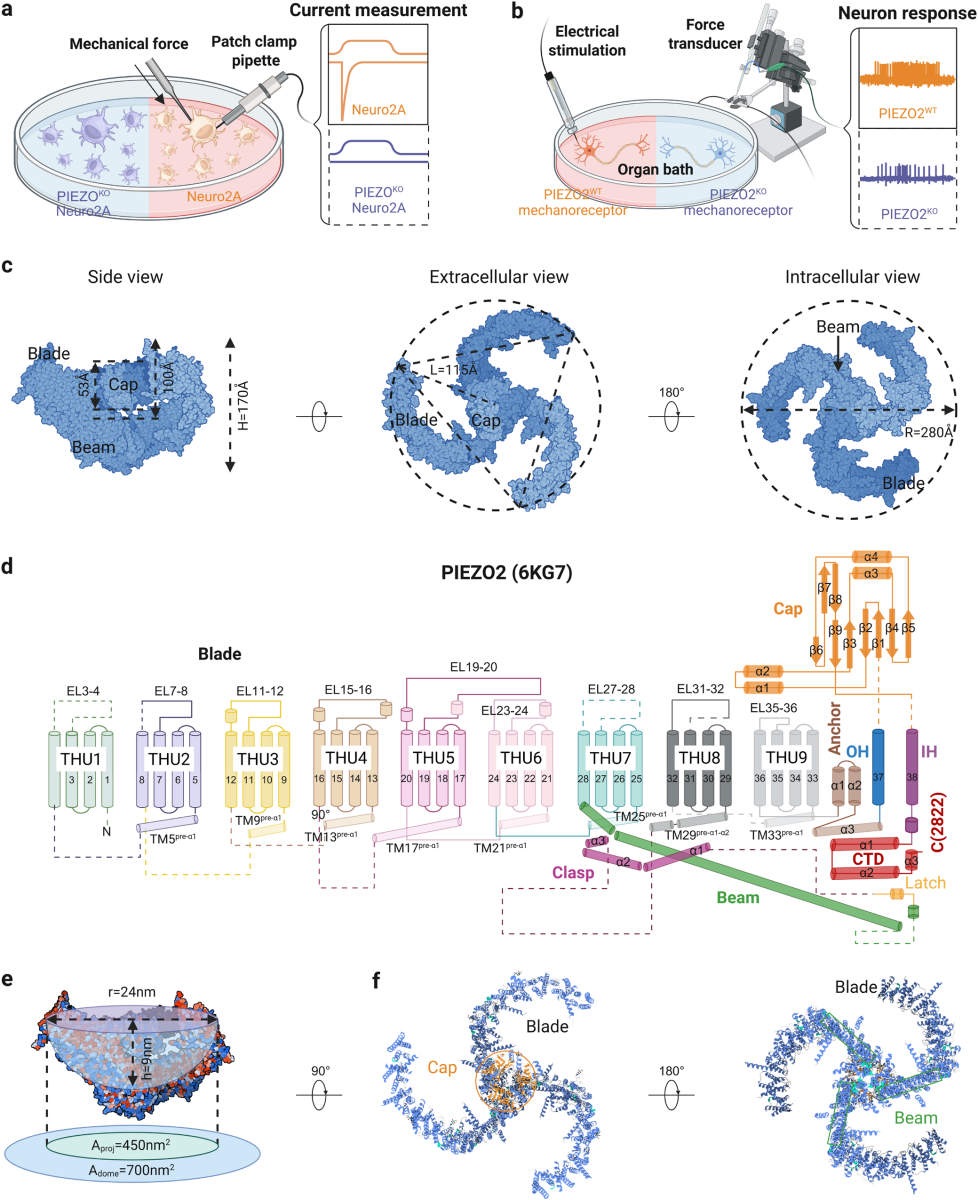
Overview of the discovery and structure–function relationship of Piezo2 channels. (a, b) Technologies used in the discovery of PIEZO channel family. The mechanically activated (MA) ion channel Piezo1 was identified using small interfering RNA‐mediated genes silencing and patch‐clamp electrophysiology. Sequence functional identification was performed using force transducer‐equipped probes to measure mechanical thresholds for nociceptors mediated by the homologous gene, Piezo2, in sensory neurons following electrical stimulation; (c) homotrimeric structure of Piezo2 with a resolution of 3.6–3.8 Å. The intracellular, side and extracellular views of the Piezo2 structure are shown and the size of the structure is labelled; (d) topological model of a Piezo2 protomer highlighting key functional domains (PDB: 6KG7). Unresolved regions are indicated by dashed lines. Key structural elements: EL, extracellular loop; IH/OH, inner/outer helix of the ion‐conducting pore; THU, transmembrane helical unit; (e) side view of electrostatic surface potential, ranging from negative (red) to positive (blue). The nano‐bowl region enclosed by the three non‐planar blades is illustrated in the form of a purple bowl, the midplane diameter (*r*), depth (*h*), mid‐plane dome surface area (Adome, blue), projected in‐plane area (Aproj, green) of the nano‐dome (purple bowl) are labelled; (f) cartoon models with the major structural domains labelled; created in BioRender.

The discovery of the PIEZO channel family has revolutionised our understanding of MA cation channels, opening a new era in understanding their physiological significance and molecular mechanisms. In 2010, using small interfering RNA (siRNA)‐mediated candidate gene silencing and whole‐cell patch‐clamp electrophysiology to record the MA cationic current, Piezo1 (also known as Fam38A) was identified as being responsible for mediating endogenous MA currents in neuroblastoma (Neuro2a) cell lines [10, 11]. Sequence analysis highlighted that the homologous gene, Piezo2 (Fam38B), specifically mediates MA cationic currents with rapid inactivation kinetics in primary sensory neurons, particularly in dorsal root ganglia (DRG) (Figure 1a,b). Heterologous overexpression of Piezo1 or Piezo2 induces two kinetically distinct MA currents in various cell types [8, 10, 11]. The cryo‐electron microscopy structure of mouse Piezo2 was determined in 2019, revealing its three‐bladed, propeller‐like homotrimeric architecture with a diameter of 280 Å and a height of 170 Å comprising 114 transmembrane helices (38 per protomer) (Figure 1c,d). The first 36 transmembrane helices (TM1‐36) are folded into nine tandem transmembrane helical units to form three non‐planar blades that collectively curve into a 9‐nm deep nano‐dome (outlined by the approximated sphere in Figure 1e), whereas the Cap, outer helix TM37 and inner helix TM38 trimerise to form the extracellular central ion‐conducting pore module [12]. Given the demonstrated membrane‐curvature induction by reconstituted Piezo1 in liposome membrane, PIEZO2 also exerts similar residing membrane‐deforming effects, producing a projected in‐plane area (*A*_proj_) of 450 nm^2^ and a mid‐plane dome surface area (*A*_dome_) of 700 nm^2^ [12, 13] (Figure 1e,f). Remarkably, the pore module features both transmembrane and cytoplasmic constriction sites, suggesting a mechanogating mechanism conserved in PIEZO channels [12, 14, 15]. Moreover, the 9‐nm‐long intracellular beam structurally bridges the blade and pore module, thereby enabling the blade‐beam module to achieve long‐distance mechanical or chemical activation of Piezos [9, 12].

**FIGURE 2:**
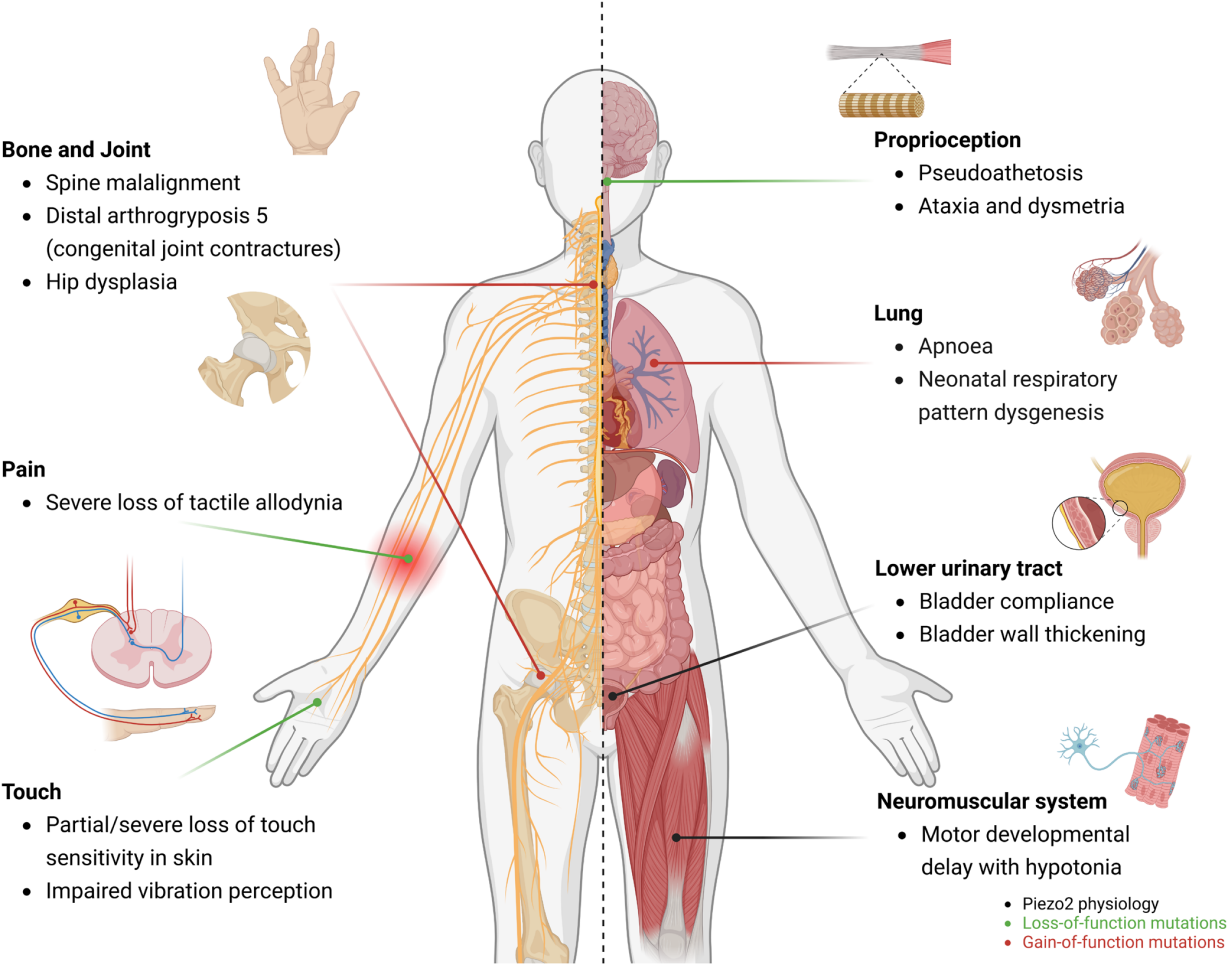
Piezo2‐mediated physiology and human genetic diseases. PIEZO2‐mediated mechanotransduction is indispensable for the physiological functions of specialised cell populations and multiple organ systems. Point mutations in human PIEZO2 are identified in both somatosensory and skeletal disorders. Phenotypes associated with loss‐of‐function mutations are indicated by the green arrow, while those associated with gain‐of‐function mutations are indicated by the red arrow; created in BioRender.

This specialised structural design of Piezo2 channel proteins facilitates force‐induced deformation and unique curvature‐based gating, enabling the precise conversion of diverse mechanical stimuli into specific signalling events and systemic physiological responses. Subsequent studies involving Piezo2 knockout mice and patients with PIEZO2 loss‐of‐function variants have established its critical roles in touch sensation and proprioception [16, 17, 18], pulmonary stretch [19], brain‐gut interactions [20, 21] and urination [22], as well as in various critical physiological systems. Conversely, PIEZO2 gain‐of‐function mutations in humans are clinically associated with distal arthrogryposis 5, characterised by congenital joint contractures, ophthalmoplegia and restrictive lung pathology [6, 23, 24] (Figure 2). This review focuses on recent advances in elucidating the biophysical properties of Piezo2 channel proteins and their crucial role in maintaining physiological homeostasis in the human body. The specific endogenous molecules as well as the types of sensory neurons that influence Piezo2 subtype‐specific modulation are also discussed. As the precise mechanotransduction mechanism of Piezo2 is explored further, new insights into the pathogenesis of human diseases and novel therapeutic interventions are anticipated.

## 2 Piezo2 Physiology: Mechanosensory Modalities and Phenotypes

### 2.1 Mechanosensation

Mechanosensation, which is the cellular transduction of mechanical forces into electrochemical signals, constitutes a fundamental biological mechanism that enables adaptive responses to external and internal stimuli in humans and other organisms [1, 2]. Recently, multiple functionally and anatomically distinct classes of somatosensory neurons have been identified, but the comprehensive molecular profile by which perceived sensory inputs combine to evoke the sense of touch remains unclear. Similarly, proprioception is considered essential for movement and postural control, but little is known about the underlying mechanisms and the precise role of this sense [25, 26].

#### 2.1.1 Touch

As accumulating studies have focused on the MA ion channel Piezo2, its relationship with touch sensation has garnered considerable attention, providing a new perspective for the development of mechanosensory responses. With siRNA‐mediated knockdown and overexpression in DRG neurons, Piezo2 was found to specifically mediate rapidly adapting (RA) MA currents linked to touch [11]. Notably, constitutive Piezo2 deletion in mice resulted in perinatal lethality owing to breathing deficits, and intrathecal injection of Piezo2 siRNA had no effect on Piezo2 transcript levels in the DRG [19, 27]. Therefore, conditional gene knockout (CKO) strategies have been employed to examine Piezo2 function.

Interestingly, AdvillinCre; Piezo2^CKO^ (Piezo2^iAdv^) mice, which exhibited postnatal Piezo2 knockout in peripheral neurons, demonstrated specific defects in innocuous touch sensations, such as those elicited by cotton swab stimuli and static light touch (von Frey test). Notably, there were no differences in response thresholds to noxious mechanical or thermal stimuli between control and Piezo2^iAdv^ mice, suggesting that Piezo2 primarily functions in response to innocuous mechanical stimuli. Furthermore, ex vivo skin‐nerve recordings revealed that, despite a significant loss of mechanosensitivity, the axonal conduction velocities of Aβ‐(touch sensation), Aδ‐(fast punctate) and C‐(blunt pain) fibres nociceptors of Piezo2^iAdv^ mice were comparable to those of controls [28]. Additionally, in vivo calcium imaging of primary sensory neurons following brushing, pinching, heat and vibration indicated that adeno‐associated virus‐Cre‐mediated Piezo2 ablation completely abolished the neural response to gentle dynamic touch [29]. Through the integration of single‐cell RNA sequencing and in vivo Ca^2+^ imaging, the study further demonstrated complete gene silencing in both slowly adapting type 1 (SA1) and RA Aβ low‐threshold mechanoreceptors (LTMRs) following Piezo2 knockout, two primary functional classes of Aβ LTMRs which only responded to gentle stimuli [30]. Recently, HoxB8Cre; Piezo2^KO^ (Piezo2^HoxB8^) mice exhibited more complete ablation of Piezo2 in DRG neurons, providing a novel and valuable tool for in vivo analysis of Piezo2 function in mechanosensory responses. Unlike Piezo2^iAdv^ mice, which displayed specific defects only in static light touch, Piezo2^HoxB8^ mice displayed severely diminished responses to innocuous mechanical stimuli [31]. Collectively, these studies established Piezo2 as the primary molecular sensor mediating the detection of various dynamic, punctate and repetitive non‐noxious mechanical stimuli.

It is well established that the Piezo2 channel is predominantly expressed in DRG neurons as well as some cutaneous mechanoreceptors and exerts important effects on the transduction and encoding of touch stimuli [32, 33], particularly the interactions between Merkel cells and SA1 Aβ neurons [34, 35]. Owing to the vital role of Atoh1 in Merkel cell development, epidermal‐specific Atoh1 conditional knockout (Atoh1^CKO^) mice completely failed to develop Merkel cells in touch domes and footpads, while maintaining otherwise normal epidermis [32, 36, 37]. Notably, ex vivo recording behavioural assays reported that, in a remarkable parallel to Atoh1^CKO^ mice, epidermal‐specific Piezo2^CKO^ mice exhibited similar deficits in SA1 firing and a reduction in behavioural responsiveness to gentle touch, indicating the physiological role of Piezo2‐dependent Merkel cell signalling in mechanosensation [16, 38]. Furthermore, the Piezo2‐mediated transduction of sensory stimuli into Ca^2+^‐action potentials may represent a potential molecular mechanism for transducing and encoding tactile stimuli to drive important tactile afferents (SA1) in Merkel cells [39]. Recent research has indicated that the Piezo2 channel is specifically activated by low‐threshold positive pressure and is highly regulated by microtubules in Merkel cells, thereby providing novel insights into the increasingly sophisticated understanding of the roles of Piezo2 in light‐touch sensation [40, 41]. While it is evident that Piezo2 is essential for touch transduction, further studies are required to elucidate the precise contribution and distribution of Piezo2 in each sensory neuron.

#### 2.1.2 Proprioception

In parallel with the substantial body of research focused on the biological functions of Piezo2 in touch perception, considerable attention has also been directed towards proprioception. Traditionally, touch perception and proprioception have been viewed as relatively independent components of the somatosensory system. However, recent studies have provided compelling evidence that these two processes are functionally interconnected [42, 43]. Emerging perspectives suggest that innervate muscle spindles (MSs) and Golgi tendon organs (GTOs) contribute to proprioception, encompassing position sense, velocity and force [44].

Based on both clinical observations and animal data, severe proprioceptive deficits appear to be among the most profound phenotypic consequences of the absence of Piezo2 function. Interestingly, this finding aligns with those of studies in mice demonstrating that Piezo2 is abundantly expressed in the sensory endings of proprioceptors, particularly in the MSs and GTOs, and the lack of Piezo2 leads to a dramatic decrease in both RA currents in DRG and stretch‐sensitive neuronal activity in proprioceptive muscle afferents [18]. Notably, two independent mouse lines lacking Piezo2 in proprioceptive neurons (PvalbCre; Piezo2^CKO^ mice and Piezo2^HoxB8^ mice) exhibited severely impaired limb coordination in the absence of obvious structural and quantitative changes in proprioceptor sensory endings, underscoring the fundamental role of Piezo2 in proprioceptive mechanotransduction [18]. Moreover, the mesencephalic trigeminal nucleus neurons represent a homogeneous population of proprioceptor neurons involved in processing proprioceptive information from the oral cavity and face. Evidence further demonstrates that Piezo2 has the potential to fulfil critical roles in RA mechanosensitive currents in these neurons, highlighting the widespread and essential function of Piezo2 in mechanotransduction in central proprioceptive neurons and, consequently, in proprioception itself [45].

While the identification of the Piezo2 channel in sensory neurons is well‐established, further investigation is necessary to comprehensively elucidate its diverse roles and mechanisms. Although the molecular transduction of proprioception by Piezo2 provides insights into the underlying mechanism of this sense, the central mechanisms responsible for these adaptations remain to be fully established. For example, while the majority of Piezo2‐dependent proprioceptive neurons express RA currents, there are also reports of a slowly adapting pattern of afferent discharge [16]. Consequently, further research is required to clarify the discontinuity between Piezo2, RA transduction currents and SA firing.

### 2.2 Nociception and Somatosensory Aberrations

The somatosensory system relies on a diverse array of signals ranging from light touch to pain and itching. Touch is crucial for communication and spatial awareness. However, innocuous mechanical stimuli induced by lesions or diseases can result in neuropathic pain and itching [46, 47]. Pain and itch are distinct sensations, and recent studies have identified multiple overlapping neural pathways and molecular mechanisms involved in both somatosensory aberrations [48, 49]. In addition to being expressed in LTMRs, Piezo2 is also detected in several classes of pain‐sensing neurons (nociceptors) [50, 51] highlighting its potential roles in pain and itch sensations.

#### 2.2.1 Pain and Mechanical Allodynia

Although it is widely acknowledged that the Piezo2 channel acts as a transducer for touch sensation and proprioception, there are also reports that mechanical nociception is partially dependent on this ion channel. Optogenetic activation of Piezo2+ somatosensory neurons can mediate MA currents in nociceptors and induce various nocifensive behaviours such as vocalisation, paw licking, flinching and jumping [31]. Of note, although ex vivo skin nerve preparation studies found reduced firing frequency of Aδ‐ and C‐fibres, Piezo2^HoxB8^ mice showed a significantly reduced behavioural response to innocuous mechanosensation and similar, but less severe, deficits in noxious mechanosensation [31]. Moreover, individuals with loss‐of‐function variants in Piezo2 also exhibit normal response thresholds to noxious mechanical stimulation, indicating a minor role of Piezo2 in the detection of certain acute painful stimuli [29] (Table 1).

**TABLE 1:** Overview of the current discovery landscape in the field of PIEZO channels.

	Piezo1/PIEZO1	Piezo2/PIEZO2
Architecture of Piezo channel	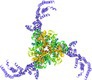	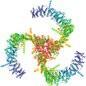
Structural architecture	2547 residues	2822 residues
Pressure of half‐maximal activation	25–30 mmHg	~50 mmHg
Inactivation kinetics	Both rapidly/slowly inactivating MA channel	Rapidly inactivating MA channel
Agonist	Yoda1/2 and Jedi1/2	Not reported
Antagonist	GsMtx4, ruthenium red, gadolinium ions, Dooku1 (competitively blocks Yoda1), and so forth	
Expression pattern	Ubiquitous functions in many different cell types	Restricted expression mainly in primary sensory neurons
Human channelopathies	LOF: Generalised lymphatic dysplasia; Prune belly syndrome GOF: Hereditary xerocytosis; Malaria resistance; Iron overload; Increased physical performance	LOF: Peripheral sensory dysfunction; Abnormal proprioception behaviour GOF: Distal arthrogryposis; Gordon syndrome; Marden‐Walker

Mechanical allodynia, a prominent symptom of neuropathic pain following inflammation or nerve injury, refers to a painful sensation that is characteristically evoked by innocuous somatosensory stimuli such as light touch [50, 52]. Interestingly, in vivo Ca^2+^ imaging of Piezo2^CKO^ neurons revealed that Piezo2 knockout is involved in the attenuation of tactile allodynia induced by spared nerve injury (SNI), as well as chronic inflammation following the administration of complete Freund's adjuvant or capsaicin [29]. Furthermore, neither brush‐evoked allodynia nor a reduced mechanical threshold in response to capsaicin‐induced inflammation and SNI was observed in Piezo2^HoxB8^ and Piezo2^iAdv^ mice [31]. Importantly, individuals with Piezo2 loss‐of‐function variants consistently reported gentle stimuli surrounding capsaicin‐inflamed skin as unpleasant and painful, highlighting the potential role of Piezo2 channel inhibition as a promising intervention for tactile allodynia and the associated pain [29]. A recent study in knock‐in mice revealed that gain‐of‐function mutations (R2756H/K) disrupt voltage‐dependent inhibition, sensitising Piezo2 channels to lower mechanical thresholds and inducing a few nociceptors to exhibit hypersensitive MA currents and ongoing activity, even in isolated neurons. This voltage‐block dysregulation drives Piezo2‐mediated nociceptor hyperexcitability, demonstrating that pathological membrane depolarisation may directly sensitise pain pathways by unlocking Piezo2's voltage‐dependent inhibition [53].

Although mechanical allodynia is a common symptom of neuropathic and inflammatory pain, the underlying molecular mechanisms by which Piezo2 contributes to mechanical allodynia, including its final effectors and neuronal subtypes, have not been widely studied. Notably, upregulated Piezo2 activity can be induced by the activation of protein kinases A and C, leading to Piezo2‐dependent RA channel activity and inflammatory mechanical allodynia [27]. Additionally, endophilin A2 (EndoA2) regulates mechanical pain sensitivity by forming a KIF5B/EndoA2/Piezo2 complex, which is essential for Piezo2 membrane trafficking in sensory neurons, thereby maintaining touch and mechanical hypersensitivity [54]. Stomatin‐like protein‐3 (STOML3), a protein facilitating force transfer and tuning the sensitivity of the Piezo2 channel, was found to directly enhance the sensitivity of mechanotransduction in injured sensory afferents owing to nerve injury or inflammatory neuropathies [55, 56, 57]. In recent years, studies have revealed an interaction between exchange proteins directly activated by cyclic AMP (Epac1)‐Piezo2 axis and mechanical allodynia during neuropathic pain. Potentiation of Epac1 signalling was reported to selectively activate Piezo2‐mediated mechanotransduction, contributing to long‐lasting allodynia [58, 59]. Emerging evidence further demonstrated that a significant increase in cyclic AMP level as well as Epac1‐dependent Piezo2 potentiation could be induced through activation of adenosine receptors (P1 receptors) and P2Y purinergic receptors by adenosine 5′‐triphosphate (ATP), which then stimulates mechanical stress evoked Ca^2+^ influx [59]. Here, the positive feedback loop between Ca^2+^ influx and Piezo2 plays a pivotal role in the pathogenesis of mechanical allodynia [59]. Furthermore, peroxisome metabolism may represent another underlying mechanism pertinent to the pathophysiology of Piezo2‐mediated mechanical allodynia. In mice deficient in the peroxisomal half‐transporter ABCD1, persistent mechanical allodynia was observed beginning at 8 months of age due to an increase in *Piezo2* gene expression in DRG neurons, along with the accumulation of saturated very‐long‐chain fatty acids (VLCFAs) in satellite glial cells [60]. Notably, the perturbation of VLCFA metabolism might further impact Piezo2‐dependent allodynia, as tension within the lipid bilayer activates the Piezo2 mechanotransduction channel through conformational changes and gating movements [60, 61, 62].

These mouse and human genetic studies provide novel insights and opportunities to explore the mechanobiology of hypersensitivity and develop novel analgesics for treating aberrant pain perception. However, further research is required to elucidate the detailed mechanisms linking ion channel dysfunction to human health and disease.

#### 2.2.2 Mechanical Itch and Alloknesis

Chronic mechanical itch, also known as alloknesis, is a type of itch evoked by mechanical light stimulation and is associated with ageing and various pathological conditions, including skin disorders and systemic diseases [63]. The underlying mechanisms involve the release of endogenous pruritogens from immune cells, overactivation of sensory neurons in the skin and alterations in central nervous system activity.

Recent studies have revealed the role of Merkel cell‐neurite complexes in mechanical alloknesis. Notably, studies have identified a pathological transition of Piezo2 channel‐Merkel cell signalling from protecting against mechanical itching under physiological conditions to a promotive role in alloknesis [17, 64]. Mas‐related G protein‐coupled receptors (Mrgprs) in primary sensory neurons have emerged as critical components in promoting itch transduction, supporting the existence of itch‐specific neuronal pathways [65] MrgprA3^+^ C pruriceptors have been reported to migrate towards Merkel cell‐enriched touch domes in the context of dry skin [64]. A recent study demonstrated that the phospholipase C‐protein kinase C δ signalling‐mediated activation of TRPV1^+^/MrgprA3^+^ DRG neurons results in the sensitisation of Piezo2 channel function on Merkel cells, thereby significantly inducing mechanical itch [66]. Conversely, Merkel cell‐specific Piezo2 knockout mice exhibit sensitised mechanical itch behaviours, and age‐related allokinesis may result from declining Merkel cell populations in aged skin [17]. Another study reported that Piezo2 is expressed in a small subset of somatostatin^+^/natriuretic polypeptide precursor B 1^+^ sensory neurons and MrgprD1^+^ C‐fibre polymodal nociceptors, which are required to transmit chemical itch and mechanical pain, implying their possible involvement in mechanical itch transmission [67]. Nevertheless, no differences in itch sensitivity have been reported in PIEZO2 deficiency syndrome. Further research is therefore necessary to develop new concepts and unearth discoveries regarding the physiological mechanisms of itching.

## 3 Piezo2 Pathophysiology: Mechanistic Insights Into Multiple Organ Systems and Associated Disease Manifestations

Upon activation, Piezo2 serves as a critical mediator of mechanosensory perception and subsequent signal transduction. A growing body of in vivo and in vitro research demonstrated that dysfunction of this channel can have significant pathophysiological consequences. The organ‐specific pathological manifestations of these sensory modalities as well as the underlying molecular mechanisms have been described below, and the summary is presented in Table 2.

**TABLE 2:** Summary of physiological functions and associated diseases of Piezo2 in various organs.

Organ systems		Cell types	Animal models	Physiological functions	Relevant diseases	References
Neuroskeletal system	Bone	Bone mesenchymal; osteoblast progenitor cells	*PValb* ^*Cre*^ *; Piezo2* ^*fl/fl*^ mice; *Prrx1* ^*Cre*^ *; Piezo2* ^*fl/fl*^; *Piezo1/2 DKO* mice	Mechanotransduction essential for skeletal development and homeostasis	Skeletal abnormalities (scoliosis and hip dysplasia)	[68, 69]
	Joint	Primary chondrocytes	*Na*_*V*_ *1.8::GCaMP6s* ^*fl/+*^ mice; *Piezo2* ^*fl/+*^ mice	Sensing and translating noxious mechanical loadings; mechanical sensitization in inflammatory OA	OA; OA chronic pain	[70, 71, 72, 73, 74, 75]
Craniofacial system and dentistry	Pulpal axon	Dental primary afferent neurons	Not reported	Transducing dentinal fluid movement and facilitating nociceptive signalling	Dental pain	[76, 77]
	PDL	PDL stem cells; apical mesenchymal cells	*Trpv1* ^*Cre*^ *; Piezo2* ^*fl/fl*^ mice; *Gli1‐Cre* ^*ER*^ *; Fgfr1* ^*fl/fl*^ mice	Orthodontic tooth movement and alveolar bone remodelling; tooth root development/regeneration	Not reported	[78, 79, 80, 81]
Lungs		NEBs	*Piezo2* ^*−/−*^ mice; *Tie2* ^*Cre*^ *; Piezo* ^*fl/fl*^ mice; *Piezo2‐ires‐Cre; Nkx2.1‐Flpo* mice	Airway mechanotransduction; normal respiration regulation (lung volume regulation and the Hering–Breuer reflex)	Respiratory distress (newborn); respiratory diseases (adult)	[19, 82]
GI tract		ISCs; EECs; organoid model of GI epithelia	*Villin‐creERT2; Piezo* ^*fl/fl*^ mice; *Trpv1* ^*Cre*^ *; Piezo2* ^*fl/fl*^ mice; *Nav1.8‐Cre* ^*+/+*^ *; Piezo2* ^*fl/fl*^ mice	Gastrointestinal mechanosensation	Colonic hypersensitivity	[20, 83, 84, 85]
		EECs; QGP‐1 cells	*NeuroD1* ^*cre*^ *; Piezo2* ^*fl/fl*^ mice *Villin1* ^*cre/+*^ *; Piezo2* ^*fl/fl*^ mice	Serotonin release and intestinal secretion; intestinal motility	Not reported	[86, 87, 88, 89, 90, 91]
Bladder		Umbrella cells	*Upk2* ^*cre*^ *; Piezo2* ^*fl/fl*^ mice; *Hoxb8* ^*cre*^ *; Piezo2* ^*fl/fl*^ mice; *Piezo1/2 DKO* mice	Urothelial mechanotransduction; bladder‐stretch sensing and urethral micturition reflexes	Deficient bladder‐filling sensation; circadian‐dependent urinary incontinence	[22, 92, 93, 94]
Cochlea		HCs; cochlear organoids	*Pax2* ^*Cre*^ *; Piezo2* ^*fl/fl*^ mice; *Atoh1* ^*Cre*^ *; Piezo2* ^*fl/fl*^ mice	Hair‐bundle mechanotransduction; sensory HC generation	Auditory defects	[95, 96, 97, 98]
Other physiological systems	Adipose tissue		*PIEZO2* ^*E2799del*^ *gain‐of‐function* mice; *PV* ^*Cre*^ *; Piezo2* ^*fl/fl*^ mice	Ensuring energy homeostasis	Obesity	[99, 100]
	Sexual organ		*Scn10a* ^*cre*^ *; Piezo2* ^*fl/fl*^ mice; *Hoxb8* ^*cre*^ *; Piezo2* ^*fl/fl*^ mice	Touch‐evoked erection reflex; physiologically sexual responses	Genital hyposensitivity	[101, 102]
		Microvascular endothelial cell	*Tek* ^*cre*^ *; Piezo2* ^*fl/f*l^ rats	Vascular permeability and remodelling; sexual‐dimorphic blood pressure regulation	Hypertension	[103, 104, 105]

### 3.1 Neuroskeletal System

Mechanical loading constitutes a fundamental determinant of skeletal morphogenesis and adaptive bone remodelling. However, the molecular mechanisms underlying the transduction of mechanical stimuli into the biological signals that regulate bone formation are poorly understood. Emerging evidence has identified Piezo2 as a critical molecular transducer that bridges biomechanical stimuli with transcriptional regulation of bone and joint formation.

#### 3.1.1 Bone

While Piezo1 plays important roles in the control of bone mass and mechanotransduction, studies have highlighted Piezo2 as an additional partner of Piezo1 in the regulation and maintenance of skeletal development and homeostasis [4]. Piezo2 exhibits expression in osteoblasts, osteoclasts and osteocytes, albeit at relatively low levels [106, 107]. Notably, the expression of Piezo2 mRNA exhibited a significant negative correlation with age in both mouse cortical bone and human bone mesenchymal stem cells, where it may be involved in the regulation and maintenance of skeletal development and homeostasis [68]. In addition, the elimination of Piezo2 in proprioceptive neurons in mice caused skeletal abnormalities that manifested as hip dysplasia and spine malalignment, which were also observed in individuals with PIEZO2 deficiency syndrome [6, 69] (Figure 2). However, somatosensory overactive Piezo2 could lead to tendon deficits and severe joint contractures in mice, potentially through increased activity within the peripheral nervous system as well as exocytosis [23]. Furthermore, the deletion of Piezo2 in osteogenic or chondrogenic lineages did not result in skeletal abnormalities in mice [69].

Although the regulatory effects of Piezo2 in touch and proprioception are well documented, relatively little is known about the importance and identity of such MA ion channels in skeletal muscle homeostasis and their dysregulation during development.

#### 3.1.2 Joint

Excessive mechanical stress plays a central role in the pathogenesis of osteoarthritis (OA), and joint cells can sense this stress and contribute to disease progression by secreting specific bioactive molecules [108]. Articular cartilage is an avascular, aneural and alymphatic tissue that covers the joint, facilitates joint articulation and withstands various types of mechanical loads. Importantly, articular cartilage possesses a limited capacity for intrinsic healing and repair, and many neuroskeletal morbidities arise from imbalances in articular cartilage biosynthesis and degradation [109, 110]. Of note, chondrocytes, the primary component of cartilage, sense and translate mechanical loadings through MA ion channels, including Piezo1/2 and transient receptor potential vanilloid 4 (TRPV4) [70, 71]. Recent advances in skeletal genetics and molecular biology have demonstrated that Piezo2 channels are specifically involved in pathogenic remodelling of cartilage in response to hyper‐physiologic mechanical loadings. The loading magnitude and changes in environmental cues can modulate intracellular Ca^2+^ concentration through Piezo2 in chondrocytes. Mechanical compression with a strain of approximately 50% by atomic force microscopy of chondrocytes induced significant intracellular Ca^2+^ influx through the synergistic activation of Piezo1/2 channels [72]. Specifically, Piezo2‐mediated Ca^2+^ signalling was only evoked in response to injurious levels (18%) of the strain, which was inhibited by Piezo2 knockdown, suggesting its potential role in cartilage disease induced by hyperphysiological and injurious loadings [71].

Considering the extensive involvement of neuronal Piezo2 in skeletal homeostasis, sensory innervation is closely associated with OA pain and aberrant subchondral bone remodelling [111, 112]. Interestingly, a recent study demonstrated that a subset of joint nociceptors co‐express Piezo2 and Ntrk1, which was critical for osteoarthritic pain, and nociceptor‐specific Piezo2 conditional knockout mice (Na_V_1.8; GCaMP6s^fl/+^; Piezo2^fl/+^ mice) protected from OA mechanical sensitisation mediated by nerve growth factor or inflammation [73]. Similarly, Piezo2 expression in isolectin B4‐binding trigeminal ganglion neurons can be upregulated by infiltrated macrophages, which further exacerbating temporomandibular joint osteoarthritis chronic pain [74]. Moreover, decreased substrate stiffness in osteoarthritic cartilage was also reported to promote Piezo1/2‐mediated intracellular Ca^2+^ influx [75].

### 3.2 Craniofacial System and Dentistry

Mechanical loading exerts a critical regulatory function in craniofacial development; however, the influence of mechanotransduction signalling pathways on odontogenesis and osteogenesis was unknown [113]. Emerging evidence has shown the expression of Piezo2 in a subset of RYR2+ cells in the ameloblast layer, dental pulp stem cells and the jawbone, suggesting its vital role in tooth and jawbone development [78, 114, 115].

Teeth experience multidirectional biomechanical loading during function [116]. Piezo2 is broadly expressed in pulpal axons and ubiquitously distributed in small myelinated (Aδ) axons in the sensory root. In the dental pulp, Piezo2 is primarily localised within the unmyelinated axons in the peripheral pulp and dentinal tubules, suggesting its potential role as a low‐threshold mechanoreceptor that mediates pain perception from weak mechanical stimuli [76]. Additionally, Piezo2 is found mostly in medium‐ to large‐sized primary dental afferent neurons, where it transduces dentinal fluid movement and facilitates nociceptive signalling through peptidergic neurotransmitter release [77]. Therefore, the targeted application of selective Piezo1/2 blockers to these regions has therapeutic potential for alleviating dental pain caused by intrapulpal pressure or dentinal fluid movement.

Beyond the dental pulp, periodontal mechanoreceptors also contribute significantly to force detection. The periodontal ligament (PDL), a fibrous tissue connecting cementum to alveolar bone, serves a decisive role in periodontal tissue homeostasis [116]. Notably, the MA channels Piezo1 and Piezo2 exhibit ubiquitous expression patterns across odontogenic lineages, including periodontal ligament stem cells [78, 116]. A recent study further demonstrated intense immunoreactivity for both proteins in murine and human periodontal ligaments, with spatial expression patterns correlating with their mechanotransductive functions. These findings substantiate their pivotal role in force transmission and the conversion of biomechanical stimuli into intracellular signalling cascades [78, 79]. Moreover, Piezo2 was found to widely express in periodontal ligament afferents. Conditional knockout of Piezo2 in TRPV1‐lineage neurons resulted in decreased orthodontic force‐induced tooth movement and the number of osteoclasts in alveolar bone on the compression side, providing novel insights into the neuroskeletal relationship associated with orthodontic tooth movement [80]. Moreover, decreased Piezo2 channel in the tooth‐PDL‐alveolar bone complex, which led to decreased WNT signalling after the activation of FGF signalling, plays a crucial role in modulating the fate of cranial neural crest‐derived progenitor cells as well as maintaining the fibrous PDL during root development [81].

### 3.3 Lungs

Breathing is a critical physiological process that is dependent on mechanosensory signalling for interoceptive pressure detection throughout the respiratory system. Systemic Piezo2 ablation in mice induces severe respiratory distress in neonates, culminating in perinatal lethality within a day of birth [19]. Similarly, humans with congenital PIEZO2 deficiency syndrome continue to exhibit delayed head control and shallow breathing throughout infancy [6].

**FIGURE 3:**
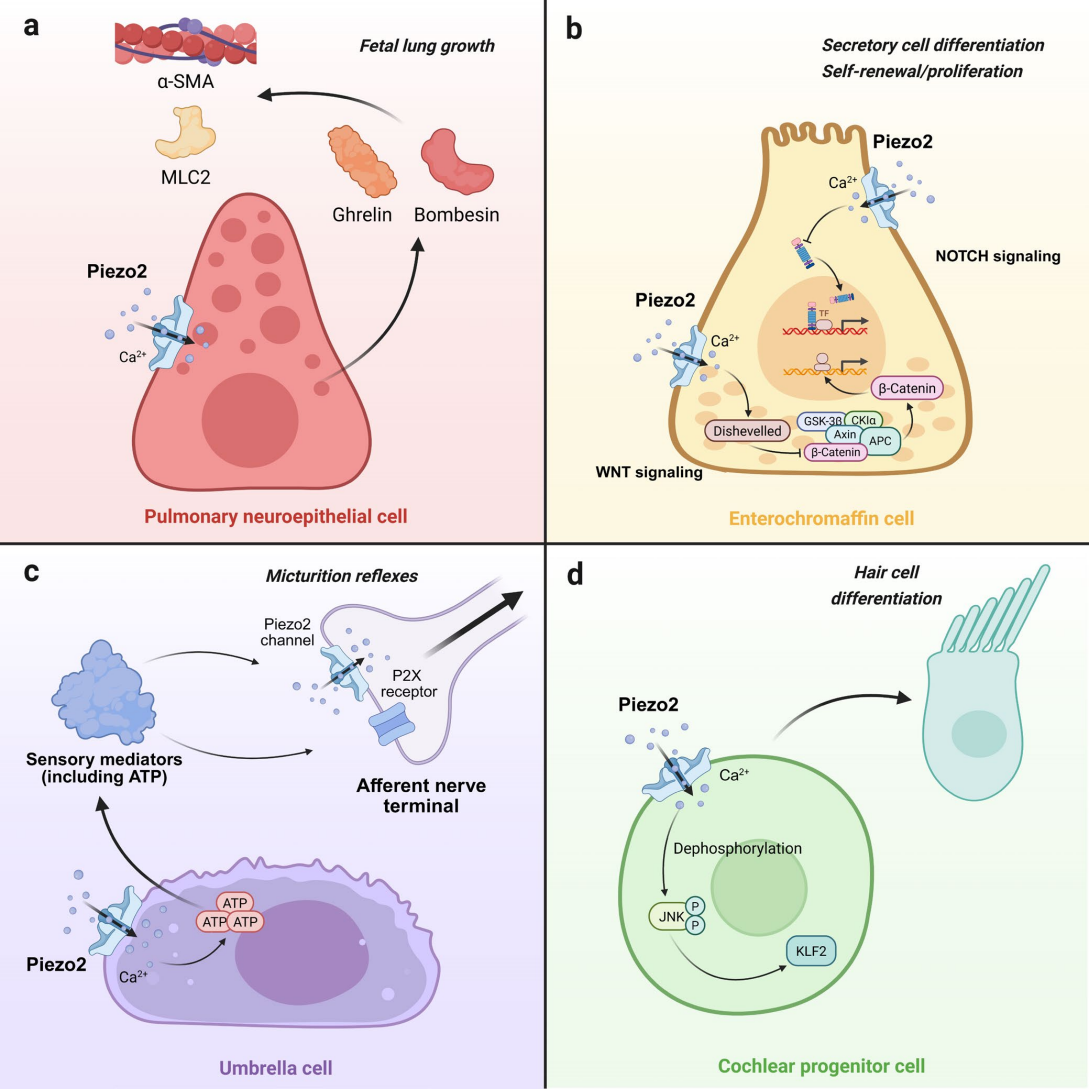
Roles of Piezo2 activation in diverse cells across physiological systems. (a) In pulmonary neuroepithelial cells, Piezo2 activation drives foetal lung growth and this effect is mediated by upregulated ghrelin, bombesin, ɑ‐SMA and MLC2 expression [117]. (b) In enterochromaffin cells, Piezo2 activation may have dual effects, with one pathway promoting secretory cell differentiation through NOTCH signalling, and the other pathway balancing self‐renewal/proliferation through WNT signalling [20]. (c) In umbrella cells, Piezo2 activation regulates both ionotropic P2X receptors and Piezo2 channels on afferent nerve terminals through secretion of signalling molecules, ultimately resulting in appropriate micturition reflexes [22, 92, 93]. (d) In cochlear progenitor cells, activation of Piezo2 channels has been observed to affect the intracellular Ca^2+^/ERK1/2/KLF2 signalling cascades, ultimately leading to hair cell differentiation [98]. Created in BioRender.

During breathing, the pulmonary system undergoes rhythmic mechanical loading with a multi‐directional stress pattern. Unsurprisingly, Piezo2 is also expressed in pulmonary neuroepithelial cell bodies (NEBs), vagal and spinal sensory neurons and several classes of endothelial cells [19, 51]. Developmental genetics demonstrated that both global and sensory neuron‐specific knockout of Piezo2 disrupts mechanosensitive signalling essential for establishing adequate respiration, resulting in complete perinatal lethality due to similar severe respiratory distress. Contrastingly, optogenetic stimulation of Piezo2+ vagal sensory neurons triggered apnea in adult mice, while mice with ablation of Piezo2 in sensory neurons exhibit aberrant neuronal signalling during lung inflation, impaired Heringg‐Breuer mechanoreflex and increased resting tidal volume [19]. Single‐cell RNA sequencing and vagal ganglion imaging revealed a specialised vagal PVALB+ neurons detecting airway compression (but not stretch) and innervating Piezo2+ pulmonary NEBs. These dedicated vagal neurons‐NEB complexes mediate airway closure responses, as evidenced by eliminated gasping in response to airway closure following NEBs/PVALB neuron ablation or Piezo2 knockout in NEBs [82]. Besides, intraluminal chloride activates pulmonary neuroendocrine cells/NEBs via Piezo1/Piezo2 to regulate ghrelin, bombesin, α‐SMA and MLC2 expression, thereby driving foetal lung growth [117] (Figure 3a). Taken together, these findings indicate that Piezo2‐mediated mechanotransduction is indispensable for the onset of pulmonary gas exchange at birth and for respiratory homeostasis throughout adulthood.

### 3.4 Gastrointestinal Tract

The gastrointestinal (GI) tract is a complex set of organs composed of various specialised mechanoreceptors, including neurons, epithelial cells, immune cells and smooth muscle cells [118]. The GI tract is responsible for oesophageal peristalsis, digestion, absorption and evacuation of waste, as well as for immunity [119]. Researches have characterised Piezo2 function in the GI tract, highlighting its importance along with endothelial cells and neurons in the control and regulation of gut sensing and motility [16, 120, 121].

#### 3.4.1 Gastrointestinal Mechanosensation

Conditional deletion of Piezo1/2 in intestinal epithelial cells (ISCs) impairs self‐renewal and lineage specification, resulting in abnormal differentiation of ISCs and secretory lineages. Mechanistically, niche stiffness/tension activates Piezo1/2 to trigger the intracellular Ca^2+^‐mediated inhibition of NOTCH signalling (promoting secretory cell differentiation) and modulation of WNT signalling (balancing self‐renewal/proliferation) [20] (Figure 3b). Additionally, Piezo2 colocalises with E‐cadherin and actin at the lateral walls of EECs, thereby forming a mechanosensitive complex essential for gut epithelial mechanotransduction. Furthermore, E‐cadherin knockdown impairs calcium responses, suggesting a conserved mechanism across epithelial mechanoreceptors in the skin, lungs and bladder [83]. Similarly, a previous study identified mechanosensitive Piezo2 channels expressed by TRPV1‐lineage nociceptors as mediators of visceral mechanosensitivity and nociception under physiological conditions and contributed to visceral hypersensitivity under pathological conditions in mice [84]. Besides, recent studies have revealed a sex‐specific role for Piezo2 in colonic mechanosensation and pain hypersensitivity, demonstrating that nociceptor‐specific Piezo2 deletion selectively impairs innocuous colonic mechanosensation in female mice, whereas intersectional Piezo2 activation exclusively induces colonic hypersensitivity in male mice [85].

#### 3.4.2 Intestinal Secretin Release

The release of intestinal secretin is regulated by several factors. Mechanical stimulation is a vital factor that can stimulate or inhibit the release of enteric secretin, which is involved in the maintenance of GI homeostasis [122, 123]. Interestingly, cell lineage tracing and superresolution microscopy confirmed the presence of Piezo2 in a subset of enterochromaffin cells (ECCs), and these channels were distributed near serotonin vesicles, indicating functional coupling between serotonin release and intestinal secretion [86]. As a specialised mechanosensor, Piezo2 activation in ECCs evokes a characteristic ionic current and increases intracellular calcium levels, resulting in mechanosensitive serotonin release [87, 88]. Meanwhile, Piezo2 inhibition via drugs or molecular knockdown decreases mechanosensitive currents, serotonin release and downstream physiological effects [86, 88]. Thus, Piezo2 may be a novel mechanosensitive factor for serotonin release.

#### 3.4.3 Intestinal Motility

Intestinal motility, which is critical for propelling intestinal contents, is driven by a rhythmic pattern of contractions and relaxations orchestrated through the coordinated interplay between smooth muscle cells and the enteric nervous system. Neuronal Piezo2 is directly associated with the sensation of intestinal contents and the modulation of peristaltic contractions, which are critical for digestion, nutrient absorption and waste removal [21, 89]. Notably, Piezo2‐dependent EEC mechanosensitivity plays an important role in adjusting contraction frequency in response to chemical environments and mechanical forces [86, 90, 124]. Single‐cell RNA sequencing and EEC lineage tracing revealed the subpopulation of Piezo2+ EECs (Piezo2^hi^ EECs) involved in neuroepithelial communication and mechanosensation [86, 90]. This cell type has been reported to express not only well‐known transcription factors and signalling molecules but also receptors and ion channels that are important in signal transduction and luminal environment sensing [90]. Furthermore, Piezo2 activation in EECs leads to the transmission of sensory information to the surrounding intrinsic primary afferent sensory neurons responsible for gut motility and intestinal secretion, thus contributing to the regulation of intestinal physiology. Moreover, a recent study discovered the specific vagal sensory feedback provided by Prox2/Runx3 neurons, which correspond to the Piezo2+ neuron subtypes innervating the upper GI tract (oesophagus and stomach) [91]. Piezo2 activation in EECs leads to the release of signalling molecules such as serotonin, substance P and peptide YY. These molecules may have additional effects on intestinal motility and warrant further investigation [89, 90]. Consequently, Piezo2 is critical for EEC mechanosensitivity and intestinal motility.

### 3.5 Bladder

The lower urinary tract, whose functions in urine storage and elimination are regulated by complex neural pathways, features a mechanosensory system in which the urothelium, historically considered a passive barrier against osmotic gradients and pathogens, is redefined as a dynamic mechanosensory interface [125]. The urothelium collaborates with mechanically sensitive afferents from the DRG to detect bladder distension through mechanotransduction pathways, thereby mediating stretching and pressure sensing during bladder filling [92, 126].

Piezo2^CKO^ DRG neurons exhibit diminished mechanosensitive responses evoked by bladder distension while maintaining normal nociceptive signalling in response to mechanical pinching, highlighting their specific role in bladder stretch sensation [22]. In addition, cystometry coupled with urethral electromyography in Piezo2‐knockout mice has revealed decreased bladder distension sensitivity accompanied by irregular micturition timing, a prolonged intermicturition interval, higher bladder pressures before micturition contractions and urethrovesical dyssynergia, collectively implicating PIEZO2 as a critical regulator of low‐threshold bladder stretch sensing and urethral micturition reflexes [22, 93]. Of note, umbrella cells establish an impermeable barrier and respond to membrane stretching through secretion of signalling molecules, with ATP emerging as the predominant messenger in bladder voiding reflexes by activating ionotropic P2X receptors on subepithelial nerve terminals [22, 92, 93] (Figure 3c). Male mice with conditional deletion of Piezo2 in urothelial cells showed urinary incontinence during the active dark phase. Dual Piezo1/2KO mice demonstrated the most pronounced phenotype, characterised by attenuated urothelial responses to mechanical stimuli, impaired ATP secretion and bladder hypoactivity in Piezo1/2 knockout females under anaesthesia. Besides, both male and female Piezo1/2‐deficient mice exhibited circadian‐dependent urinary incontinence, manifesting exclusively during the active dark phase with no leakage observed in the inactive light period [94]. These findings suggest that urothelial mechanosensation operates through sexually dimorphic and circadian‐regulated mechanisms, establishing critical functional links between Piezo1/2‐mediated mechanoelectrical transduction pathways and the maintenance of normal physiological voiding processes.

Emerging evidence also highlights the critical involvement of PIEZO2 mechanosensitive ion channels in proper urinary function in humans. PIEZO2‐deficient patients not only exhibit profound distal arthrogryposes and tactile sensory deficits but also experience bladder dysfunction characterised by decreased voiding frequency and sudden urge incontinence [6, 22]. Importantly, urinary function and associated behaviours are not entirely abolished in either Piezo2 knockout mice or patients with PIEZO2 loss‐of‐function mutations, suggesting the existence of compensatory mechanosensory pathways or partial functional preservation in specific cell populations. This partial functionality aligns with the finding that Piezo2's role becomes more prominent after tissue damage or inflammation, as observed in nociceptor‐specific knockout studies. Further research is needed to elucidate how Piezo2 coordinates micturition reflexes. Understanding this process may inform the development of therapeutic strategies for the treatment of lower urinary tract diseases.

### 3.6 Cochlea

The hearing sensory organ is one of the most complex structures in mammals, where highly specialised hair cells (HCs) serve as critical mechanotransducers. These specialised epithelial cells mediate auditory perception through sophisticated mechanotransduction processes that transform acoustic energy from environmental vibrations into electrochemical signals.

Piezo2, a mechanically sensitive ion channel protein, plays a critical role in auditory transduction by converting mechanical stimuli into electrochemical signals in the cochlea [95]. Transmembrane channel‐like (TMC) 1 and 2 reportedly localise to the site of mechanotransduction in mouse HC stereocilia and are required for its transduction channels [127, 128]. Similarly, Piezo2 is expressed in the cochlea and vestibular HCs but does not serve as an essential mechanotransduction channel in the stereocilia of outer hair cells (OHCs). Instead, Piezo2 labelling occurs in the apical region of HC bodies, with the highest density observed near the adherens junctions adjacent to the longest stereocilia, mediating reverse‐polarity currents during developmental maturation, post‐mechanical disruption of tip links and in TMC1/2‐deficient HCs [95]. Ex vivo cochlear Ca^2+^ imaging revealed the indispensable mechanotransductive role of Piezo2 in ultrasonic transduction, wherein OHCs function as effector cells through coordinated interactions with this essential molecular transducer in ultrasonic hearing [96]. Notably, the mechanoelectrical transducer (MET) channel is the entryway for the sound‐balance‐brain interface, exhibiting the biophysical and pharmacological features of MET channels, including sub‐millisecond activation time constants, weak cation selectivity and large molecule permeability [129, 130]. The Piezo1/2 subunits co‐localise and co‐assemble with Tmc1/2 and other MET complex partners, subserving a potential pathway for ion permeation in HC stereocilia and conferring MET gateway function for sound‐balance‐brain communication [97]. A recent study reported that Piezo2, an intracellular signal modulator influencing cell fate during sensory epithelial development, mediates elevated extracellular matrix (ECM) stiffness‐induced sensory HC generation via intracellular the Ca^2+^/ERK1/2/KLF2 signalling cascades [98] (Figure 3d). Piezo2 may play a pivotal role in orchestrating the development and maturation of the mouse inner ear sensory epithelium and associated transduction mechanisms. Further research into its functional interactions with key developmental regulators could advance therapeutic strategies for HC regeneration.

### 3.7 Emerging Roles in Systemic Homeostasis and Specialised Functions

Based on the emerging evidence from recent studies, the clinical significance of Piezo2 extends far beyond its mechanosensory functions. Recent research has revealed its critical involvement in multiple physiological systems (Table 2).

#### 3.7.1 Metabolic Regulation in Adipose Tissue

A growing body of research has highlighted that active mechanosensitive processes within the adipose tissue involve dynamic cellular interactions, in which Piezo2 emerges as the predominant mechanoreceptor expressed in fat‐innervating sensory neurons [99]. Systemic metabolic homeostasis is regulated by inter‐organ crosstalk, which coordinates adipose tissue thermogenesis. Piezo2+ sensory neurons in the sympathetic‐sensory feedback loop between fat and the brain may be involved in the prevention of lipid imbalances and systemic hypermetabolism [100].

#### 3.7.2 Sexual Function

Piezo2 is also functionally expressed in both the clitoral Krause's and Meissner's corpuscles [101, 102]. Piezo2 and perineal LTMRs enable exquisite awareness of the precise somatosensory perception of the genitals, demonstrating essential functionality in the touch‐evoked erection reflex and physiologically important sexual responses [131].

#### 3.7.3 Cardiovascular and Vascular Roles

Emerging evidence has delineated crucial roles of Piezo2 in nitric oxide biosynthesis, vascular permeability and remodelling and blood pressure regulation with clinical relevance to unstable blood pressure (hypertension) [103, 104]. In particular, Piezo2 exists in Fam19a4/Nts‐positive retinal ganglion cells, a unique perivascular neuronal subset that directly contacts blood vessels and ensures the formation of a stereotyped 3D vascular lattice architecture as well as visual function through direct neurovascular interactions [105].

In recent years, new concepts and discoveries regarding the mechanosensing properties of Piezo2 have dramatically improved our understanding; however, the biological properties and protective mechanisms of Piezo2 in various aspects of mechanosensation require further research for clinical assessment and application.

## 4 Novel Disease Modelling: Advances in Exploring Piezo2 Channel Mechanisms

Current researches on the mechanosensitive ion channel PIEZO2 faces various limitations, including human tissue inaccessibility, associated deficiency syndrome rarity and unresolved technological hurdles in developing reliable predictive disease models and pharmacological screening platforms. Consequently, there is an urgent need to establish innovative experimental frameworks capable of overcoming these longstanding challenges while advancing mechanistic insights and therapeutic discovery.

The integration of stem cell technology into sensory neurobiology research has emerged as a transformative approach for investigating human mechanotransduction and nociception at the cellular and molecular levels. Theoretically, induced pluripotent stem cells (iPSCs) derived from patients and healthy individuals exhibit unprecedented multilineage differentiation capacity to generate diverse human cell lineages, including sensory neurons, in vitro [132]. In 2015, researchers initially established human iPSC‐derived RA‐LTMRs through an empirically optimised multistep differentiation protocol that achieved lineage‐specific conversion within weeks, functionally identifying the central role of Piezo2 in human mechanotransduction while simultaneously elucidating the developmental pathways governing sensory neuron specification [133]. Subsequently, by overexpressing NGN2 and BRN3A during neural crest cell differentiation, iPSCs could be selectively converted into homogeneous populations of TRPM8+/Piezo2+ sensory neurons, and further studies validated the existence of similar neurons in adult human tissue [134]. Parallel work leveraging WNT/BMP4 activation in multipotent iPSC‐derived neuronal precursor cells generated human LTMR‐like neurons with Piezo2‐enriched specialised axon termini that morphologically resembled bulbous nerve endings [135].

Organoids and biomaterials have also emerged as groundbreaking techniques for studying human organ development, diseases and drug screening [136]. For example, 2D/3D organoid systems combined with bioengineered matrices and mechanical stretching platforms have demonstrated that PIEZO2 mechanosensing of the intestinal stem cell niche regulates intestinal epithelium turnover and regeneration through calcium‐dependent suppression of NOTCH signalling to induce secretory cell lineage specification, while dynamically activating the WNT pathway to maintain the balance between self‐renewal and proliferation [20]. A recent study reported an innovative approach that included the use of cochlear organoids and a mechanically tunable hybrid hydrogel system to mimic the ECM mechanical force and dynamic formation of the cochlear sensory epithelium. The study demonstrated biphasic mechanoregulation in the fate determination of cochlear progenitor cells (CPCs): elevated ECM stiffness drives CPC differentiation into sensory HCs through Piezo2‐mediated intracellular Ca^2+^ signalling and KLF2 activation, whereas moderate stiffness enhances CPC‐derived expansion of the prosensory epithelium via the activation of ITGA3/F‐actin cytoskeleton/YAP signalling [98]. Additionally, 3D magnetic hyaluronic acid hydrogels and piezoelectric nanoarrays with mechanical–electrical coupling microenvironment models offer noninvasive neuromodulation through the stimulation of primary DRG neurons. These models elucidate the Piezo2‐mediated mechanotransduction mechanisms underlying chronic pain modulation and innervated bone regeneration, highlighting the transformative potential of Piezo2 for developing regenerative medicine and tissue repair strategies [137, 138].

These technologies provide a starting point for elucidating the molecular mechanisms of Piezo2, which will be significant for the development of novel therapeutic targets. Nevertheless, further studies are still needed to develop more disease modelling and elucidate the mechanism underlying Piezo2 as a potential treatment to prevent skeletal malformations, somatosensory disorders and other human genetic diseases.

## 5 Conclusion

As the only mechanosensor family demonstrating such profound effects across diverse biological processes, the discovery of PIEZO1/2 channels and subsequent investigations have revolutionised our understanding of force‐dependent cellular signalling. While significant advances have revealed the unique structural architecture and mechano‐electric coupling mechanisms of Piezo2, the biophysical properties, tissue‐specific physiological tuning, molecular anatomy and associated pathologies in neural populations remains not yet fully elucidated. There also exist substantial challenges in translating these fundamental insights into clinical applications. Thus, substantial efforts are still required to bridge the gap between PIEZO2 and their clinical significance:

1. While Piezo2's involvement in tactile allodynia and alloknesis is well‐documented, its mechanistic contribution to acute nociceptive signalling requires rigorous clarification.
2. The molecular crosstalk governing functional synergy or redundancy in tissues where co‐expressed Piezo1/2 channels (e.g., bone, articular cartilage and intestinal epithelia) remains undefined.
3. Clinical investigations remain limited, predominantly depending on the analysis of existing multiomics data from rare patients with genetic disorders to elucidate the expression of specific genes within the PIEZO2 signalling pathway and their relevance to disease progression.
4. Given PIEZO2's widespread expression, particular attention must be paid to targeted therapeutic agents for minimising adverse off‐target effects and enhancing therapeutic efficacy.

Future studies should integrate multi‐disciplinary approaches to address these challenges. Of note, creating comprehensive cross‐tissue molecular reference maps through single‐nucleus RNA sequencing methods and conditional variational autoencoder across various systems will enhance our understanding of the cell types and gene modules underlying disease mechanisms for complex traits. Moreover, further molecular pathological studies on clinical samples, along with systematic exploration of isoform‐selective pharmacophore modelling and tissue‐restricted delivery platforms, are essential to derive conclusive insights regarding the rationale for targeting the PIEZO2 pathway and avoid significant off‐target liabilities, achieving therapeutic specificity for PIEZO2‐targeted pharmacotherapy. In conclusion, identification of the biological properties of this remarkable ion channel will provide new insights for elucidating the detailed mechanisms underlying molecular mechanotransduction and developing novel therapeutic targets.

## Author Contributions

Mengjie Wu, Liang Xie and Qianming Chen contributed to the conception of the paper. Zhebin Cheng wrote the manuscript and drew the figures. Zuping Wu edited and revised the manuscript. All authors read and approved the final manuscript.

## Conflicts of Interest

The authors declare no conflicts of interest.

## Data Availability

Data sharing not applicable to this article as no datasets were generated or analysed during the current study.
